# Upregulation of miR-142-5p in atherosclerotic plaques and regulation of oxidized low-density lipoprotein-induced apoptosis in macrophages

**DOI:** 10.3892/mmr.2015.3191

**Published:** 2015-01-13

**Authors:** RUIJIN XU, CHENGLONG BI, JIANTAO SONG, LIN WANG, CHENG GE, XINXIN LIU, MEI ZHANG

**Affiliations:** Key Laboratory of Cardiovascular Remodeling and Function Research, Department of Cardiology, Chinese Ministries of Education and Public Health, Qilu Hospital, Shandong University, Jinan, Shandong 250012, P.R. China

**Keywords:** atherosclerosis, apoptosis, mir-142-5p, transforming growth factor β2

## Abstract

MicroRNA (miR)-142-5p is a member of the miR-142 family, which have been shown to be associated with tumors, stem cells and disorders of the immune system. However, the role of miR-142-5p in atherosclerosis has yet to be investigated. In the present study, an atherosclerotic apolipoprotein E-deficient (apoE−/−) mouse model was constructed and fed a high-fat diet. The expression levels of miR-142-5p in the murine atherosclerotic plaques were detected by gene microarray analysis. In addition, an *in vitro* assay was used to determine the expression levels of miR-142-5p in human endothelial cells, smooth muscle cells and macrophages, which were treated with oxidized low-density lipoprotein (ox-LDL). Furthermore, a miR-142-5p inhibitor and mimic was transfected into cultured human macrophages, in order to observe the effects on transforming growth factor-β2 (TGF-β2) expression. The effects of co-transfection of the miR-142-5p inhibitor or mimic with TGF-β2, in human macrophages, on the rate of apoptosis was analyzed. The expression levels of miR-142-5p were 6.84-fold higher in mice with stable atherosclerotic plaques, and 2.69-fold higher in mice with vulnerable atherosclerotic plaques, as compared with the controls. Furthermore, the expression levels of miR-142-5p were upregulated in the cultured human macrophages. The percentage of apoptotic cells was lowest in the macrophages transfected with both TGF-β2 and miR-142-5p inhibitors and treated with ox-LDL. The expression levels of miR-142-5p were upregulated in the atherosclerotic plaques of the apoE−/− mice. The findings of the present study have shown that the upregulation of miR-142-5p expression may regulate apoptosis in human macrophages by targeting TGF-β2. This effect may have an important role in the progression of atherosclerosis.

## Introduction

Atherosclerosis is a chronic inflammatory and fibroproliferative disease. Effective results have not been achieved by controlling and intervening with the associated risk factors, including smoking, dyslipidemia, hypertension and diabetes (1–3). Therefore, these risk factors may not fully explain the occurrence and development of atherosclerosis and other targets of atherosclerosis require identification.

MicroRNAs (miRNAs) are small (~23 nt), non-coding RNAs that regulate gene expression at the post-transcriptional level. MiRNAs have been shown to have an important role in the development and progression of atherosclerosis (4–6). They are expressed in a tissue-specific manner and are associated with cell proliferation, apoptosis and differentiation (7,8). MiRNAs have been previously implicated in atherosclerotic plaque formation, caused by hyperlipidemia and hypertension (9,10). MiRNAs have also been directly associated with anti-atherosclerotic signals in vascular smooth muscle and endothelial cells (11). The exact mechanisms of the role of miRNAs in atherosclerosis remain to be elucidated.

MiR-142-5p is a member of the miR-142 miRNA family which have known roles in cancer, immune diseases and embryonic stem cells (12,13). However, the expression of miR-142-5p in atherosclerotic plaque and its roles in atherosclerosis are currently unclear.

In the present study, the expression levels of miR-142-5p were detected in murine atherosclerotic plaques and human macrophages. The present study also aimed to identify miR-142-5p target genes, and its effects on apoptosis in macrophages.

## Materials and methods

### Materials

The following reagents, kits, primers and cells were used and sourced from the following companies: Anti-rabbit transforming growth factor-β2 (TGF-β2) monoclonal antibody (ProteinTech Group, Inc., Chicago, IL, USA); anti-mouse GAPDH monoclonal antibody (SunShineBio, Nanjing, China); miR-142-5p primers (Exiqon Co., Copenhagen, Denmark); TGF-β2 primers (ShangDe Biomedical Engineering, Shanghai, China); total RNA extraction kit (TRIzol^®^; Invitrogen Life Technologies, Carlsbad, CA, USA); THP-1 human monocytes, smooth muscle and endothelial cells (American Type Culture Collection, Manassas, VA, USA). Other reagents used throughout the present study were obtained from the Key Laboratory of Cardiovascular Remodeling and Function Research, Qilu Hospital, Shandong University, (Jinan, China).

### Animals

Apolipoprotein E−/− (apoE−/−) mice were purchased from WeiTong LiHua Co. (Beijing, China). The animal experiments were approved by the Institutional Animal Care and Use Committee of Shandong University (Jinan, China).

### Animal model of atherosclerosis

The eight-week-old male apoE−/− mice were fed a high-fat diet, that consisted of a standard diet plus 2% cholesterol and 5% lard oil, for two weeks, following a three day standard diet. The mice were then intraperitoneally injected with 0.08% sodium pentobarbital (40 mg/kg; Beijing OuHe Technology Co., Ltd., Beijing, China) and underwent surgery. Carotid atherosclerotic plaques were induced in the mice using perivascular constrictive collars, which were placed on the right common carotid artery, as described by previous methods (14). The mice were divided into three groups (n= 12/group): Control, stable plaque, and vulnerable plaque. The mice then received a high-fat diet for a further 12 weeks. The vulnerable plaque group underwent Pisa syndrome noise interference for four weeks during the 12 week period. In brief, experimental mice were kept in a 50 ml plastic pipe with a covered end and a through hole and then subjected to 110 dB noise stimulation intensity for 30 sec (Beijing Great Wall Radio Factory, Beijing, China), every 5 min for 6 h/day. All of the mice were sacrificed by cervical dislocation and blood samples (1 ml) were obtained from the abdominal vein and stored at −80°C, until further use. Sections of the carotid arteries were cut in optimal cutting temperature compound medium, and stored in liquid nitrogen, until further use.

### Gene microarray analysis for miR-142-5p expression

The isolated carotid artery sections were removed from liquid nitrogen and the vascular peripheral tissue was placed on ice. The expression levels of miR-142-5p, in the atherosclerotic plaques, were determined using a miRNA microarray assay with rat miRNA array probes (Kangchen Bio-tech Inc., Shanghai, China).

### Cell culture and transfection

Primary human endothelial cells and human macrophages were obtained from the American Type Culture Collection (Manassas, VA, USA) and were cultured in RPMI-1640 medium (Gibco Life Technologies, Carlsbad, CA, USA) supplemented with 10% fetal bovine serum (FBS; Beijing Solarbio Science & Technology Co., Ltd., Beijing, China) at 37°C in a 5% CO_2_ atmosphere. Human smooth muscle cells were cultured in a medium of 2% FBS, 1% smooth muscle cell growth supplement and 1% penicillin/streptomycin (Xiang Bo Biological Technology Co., Guangzhou, China) under identical conditions. The media were refreshed every 2–3 days. The cells from passages 3–5 were used for the following experiments. Following culture, cell aliquots were transferred to freezing tubes with cell freezing medium and stored at −80°C overnight and then preserved in a liquid nitrogen tank until further used. Cell were seeded onto six-well plates, when cell confluence reached 70% 5 μl diethylpyrocarbonate (1:10 dilution; Beijing Solarbio Science & Technology Co., Ltd.,) of mir-142-5p inhibitor (Shanghai GenePharma Co., Ltd., Shanghai, China) was added to 250 μl Opti-MEM (Gibco Life Technologies) culture medium and incubated at room temperature for 5 min. Subsequently, 2.5 μl Lipofectamine 2000 (Shanghai Yijie Biotechnology Co., Ltd., Shanghai, China) was added to 250 μl Opti-MEM culture medium and incubated at room temperature for 5 min. The liposome suspension was then added to the mir-142-5p inhibitor liquid and incubated at room temperature for 15 min. The suspension was then added to the cells and incubated for 6 h, the medium was then replaced and the cells were cultured for a further 48 h. To study the miR-142-5p expression levels, the cells were divided into two treatment groups: Control and oxidized low-density lipoprotein treated (ox-LDL; 90 μl, 50 mg/ml, for 24 h). To study TGF-β2 expression levels in the macrophages, the cells were divided into five groups: Control, control + ox-LDL, miR-142-5p inhibitor transfection + ox-LDL, miR-142-5p mimic transfection, and negative control (NC) + ox-LDL. To investigate the effects of miR-142-5p on the rate of apoptosis of human macrophages, the cells were divided into seven groups: Control, control + ox-LDL, miR-142-5p inhibitor transfection + ox-LDL, TGF-β2 inhibitor transfection + miR-142 -5p inhibitor + ox-LDL, miR-142-5p mimic transfection, TGF-β2 inhibitor + ox-LDL, N.C + ox-LDL.

### Quantitative polymerase chain reaction

Total RNA was extracted from the human endothelial cells, smooth muscle cells and macrophages using TRIzol^®^. A total of 1 μg RNA, from each group, was reverse transcribed using the PrimeScript RT Reagent kit (Takara Bio Inc., Otsu, Japan), and the qPCR was performed using the Bio-Rad IQ5 Real-Time PCR Detection system (Bio-Rad Laboratories Inc., Hercules, CA, USA). All reagents used for qPCR were from this detection system unless otherwise stated. The reaction system consisted of 10 μl SYRB Green Mix (Takara Bio, Inc.), 2 μl miRNA primer mix and 8 μl diluted cDNA. The following primer sequences were used: TGF-β2 forward, 5′-ACAAAATAGACATGCCGCCC-3′, and reverse, 5′-GATGGCATCAAGGTACCCACAG-3′; Hsa-miR-142-5p forward, 5′-AACTCCAGCTGGTCCTTAG-3′, and reverse, 5′-TCTTGAACCCTCATCCTGT-3′; Hsa-miR-142-5p inhibitor were, 5′-AGUAGUGCUUUCUACUUUAUG-3′; Hsa-miR-142-5p mimic forward, 5′-CAUAAAGUAGAAAGCACUACU-3′, and reverse, 5′-UAGUGCUUUCUACUUUAUGUU-3′. The housekeeping genes U6 or β-actin were used as internal controls. Primer sequences were as follows: U6 forward, 5′-CTCGCTTCGGCAGAC-3′ and reverse, 5′-AACGCTTACGAATTT -3′; β-actin forward, 5′-CGTGCGTGACATTAAGGAGA′-3′ and reverse, 5′-CACCTTCACCGTTCCAGTTT-3′. The cycling conditions were as follows: Initial denaturation at 95°C for 10 min followed by 40 cycles of 95°C for 10 sec, 56°C for 10 sec and 72°C for 30 sec. qPCR concluded with 65°C for 30 sec and 70°C for 30 sec. Changes in the gene expression levels were calculated using the cycle threshold (Ct) comparison method, by the formula 2^−ΔΔCt^.

### Western blotting

The cell lysates from the treated macrophages were prepared, as described previously (15). The Bio-Rad Protein Assay Reagent kit (Bio-Rad Laboratories Inc.) was used to measure protein concentrations. The protein samples (20 μg) were separated using 10% SDS-PAGE, at 90 V for 1 h and transferred electrophoretically to polyvinylidene fluoride membranes (Millipore, Bellerica, MA, USA), at 110 mA for 0.5 h. The membranes were then blocked with 5% milk for 2 h at room temperature, and incubated with the primary antibodies at 4°C overnight. The membranes were washed three times with tris-buffered saline containing Tween^®^ (10 min/wash), and then incubated with a secondary horseradish peroxidase-labeled antibody at room temperature for 1.5 h. The signals were visualized using an Enhanced Chemiluminescence substrate (GE Healthcare Life Sciences, Chalfont, UK).

### Apoptosis detection

The number of apoptotic human macrophages was quantified using the Annexin V-PE Apoptosis Detection kit (Beyotime Institute of Biotechnology, Hainen, China). The apoptotic cells were calculated as number of apoptotic cells/total cell number × 100%.

### Statistical analysis

The data analysis was carried out using SPSS version 17.0 (SPSS Inc., Chicago, IL, USA). The data are presented as the means ± standard error of the mean. Statistical comparisons were performed using a paired student’s t test and an analysis of variance. A P<0.05 was considered to indicate a statistically significant difference.

## Results

### Expression levels of miR-142-5p are upregulated in the atherosclerotic plaques of mice

**Figure 1:**
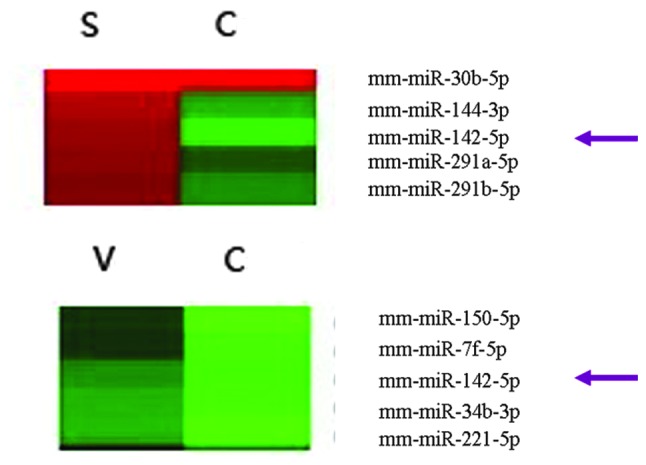
Analysis of the expression levels of microRNA (miR)-142-5p. The expression levels of miR-142-5p were upregulated in atherosclerotic plaques of apolipoprotein E-deficient mice, as determined by a miRNA microarray assay. Light green/red to dark green/red represents low to high levels of gene expression. S, tissue from mice with stable plaque in carotid arteries; V, tissue from mice with vulnerable plaque; C, normal control tissue.

The expression levels of miR-142-5p were 6.84-fold higher in the mice with stable plaques, and was 2.69-fold higher in the mice with vulnerable plaques, as compared with the controls (Fig. 1).

### Expression levels of miR-142-5p are upregulated in human macrophages treated with ox-LDL

**Figure 2:**
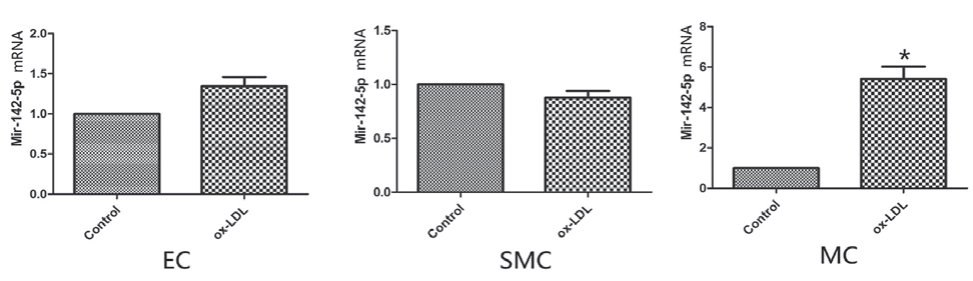
MicroRNA (miR)-142-5p was overexpressed in human macrophages. Human endothelial cells (EC), smooth muscle cells (SMC) and macrophages (MC) were treated with 50 mg/ml oxidized low-density lipoprotein (ox-LDL), for 24 h. A quantitative polymerase chain reaction was performed to determine the relative miR-142-5p mRNA expression levels, normalized to that of U6. The data are expressed as the means ± standard error of the mean from three independent experiments. ^*^P<0.05 vs control.

The expression levels of miR-142-5p in the human macrophages treated with ox-LDL were upregulated, as compared with the control macrophages (P<0.05). However, there were no marked differences from the controls in either the endothelial or smooth muscle cells (Fig. 2).

### TGF-β2 is predicted to be a target gene of miR-142-5p

**Figure 4:**
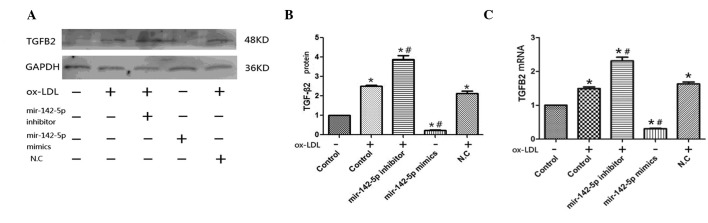
Expression levels of transforming growth factor (TGF)-β2 in human macrophages. The macrophages were transfected with mR-142-5p inhibitor or mimic, and incubated with ox-LDL for 24 h. (A) Western blot analysis of TGF-β2 protein expression levels. (B) Quantification of protein expression levels, normalized to that of GAPDH. (C) Quantitative polymerase chain reaction analysis of relative TGF-β2 mRNA expression levels, normalized to that of β-actin. The data are expressed as the means ± standard error of the mean from three independent experiments. ^*^P<0.05 vs control, ^#^P<0.05 vs control+ox-LDL. NC, negative control; KD, kilodaltons.

miRanda (www.microrna.org/microrna/home.do) target-gene prediction software was used to predict the target gene of miR-142-5p, and TGF-β2 was predicted to be the most probable target gene. To verify whether TGF-β2 was a target gene, a miR-142-5p inhibitor and mimic were transfected into macrophages. The expression levels of TGF-β2 were higher in the cells transfected with the miR-142-5p inhibitor and treated with ox-LDL, as compared with the cells undergoing ox-LDL treatment alone (P<0.05; Fig. 4), and were the lowest when the cells were transfected with the miR-142-5p mimic (P<0.05). These results suggest that TGF-β2 may be a target gene of miR-142-5p.

## Discussion

Tissue-specific expression is an important characteristic of miRNA expression (16). The present study demonstrated that miR-142-5p expression was upregulated in atherosclerotic plaques obtained from apoE−/− mice. In addition, miR-142-5p was shown to be associated with the apoptosis of macrophages, through the regulation of its predicted target gene, TGF-β2.

MiRNAs are small, non-coding, highly conserved RNAs that regulate gene expression at the posttranscriptional level (17–19). MiRNAs may negatively regulate gene expression either by promoting the decomposition of mRNAs, or inhibiting the translation of protein (20,21). MiRNAs have been shown to have an important role in cardiovascular diseases, including atherosclerosis (22–24), and can regulate the functions of endothelial cells, macrophages and vascular smooth muscle cells (25–28). They have previously been demonstrated to modulate every stage of atherosclerosis, by different stimuli (29–31).

MiR-142-5p is a member of the miR-142 family, which is involved in the pathogenesis of various diseases (13,32,33). Previous research into miR-142-5p has mainly focused on its associations with tumors, immune diseases and stem cells (32); however, its role in atherosclerosis remains unknown. In the present study, an atherosclerotic plaque apoE−/− mouse model was generated and the expression levels of miR-142-5p were upregulated in the atherosclerotic plaques of the apoE−/− mice.

Atherosclerosis is a chronic non-resolving inflammatory disease. Monocytes/macrophages are major immune cells, which are thought to be responsible for the development of atherosclerosis (34–36). In the present study, significant miR-142-5p expression was detected in macrophages, but not endothelial or smooth muscle cells. Apoptosis of macrophages has been shown to contribute to both early and advanced atherosclerosis (37,38). The accumulation of apoptotic macrophages leads to secondary necrosis, necrotic core enlargement and plaque instability (39,40). Furthermore, macrophages have been shown to be involved in cell apoptosis in atherosclerotic plaques, through targeting specific control genes (40,42). The present study determined that apoptosis of macrophages could be affected by miR-142-5p.

MiRNAs negatively regulate the expression of target genes. A database-based target gene prediction software predicted that TGF-β2 was the most probable target gene of miR-142-5p. To verify whether TGF-β2 was the target of miR-142-5p, an inhibitor and a mimic of miR-142-5p were transfected into macrophages, and the effects were observed on TGF-β2 protein and mRNA expression levels. The results verified that TGF-β2 was the likely target gene of miR-142-5p. TGF-β2 is a cytokine associated with a variety of functions, it has previously been shown to participate in cell proliferation, apoptosis and differentiation (42–45). It has an important role in the pathophysiological processes of tissue repair, inflammation, arterial atherosclerosis and cancer (45–48). In the present study, miR-142-5p was shown to be associated with the apoptosis of macrophages by negatively regulating TGF-β2. In conclusion, miR-142-5p was shown to be involved in atherosclerosis in mice, and TGF-β2 was identified as its target. MiR-142-5p was shown to regulate macrophage apoptosis by targeting TGF-β2. The present study provides a novel target for further study of atherosclerosis.

## Figures and Tables

**Figure 3:**
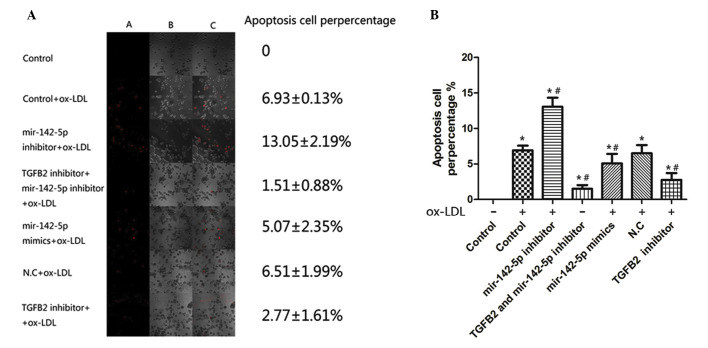
(A) MicroRNA (miR)-142-5p inhibited apoptosis of macrophages. The macrophages were transfected with a miR-142-5p inhibitor or mimic and/or transforming growth factor β2 (TGF-β2) inhibitor, and incubated with oxidized low-density lipoprotein (ox-LDL) for 24 h. Red colouration indicates apoptosis. A, Annexin V-phycoerythrin staining of red fluorescent map; B, macrophages *in situ*; C, A and B combined. (B) Quantification of the percentage of apoptotic cells. The results are expressed as the means ± standard error of the mean. ^*^P<0.05 vs control, ^#^P<0.05 vs control+ox-LDL.
